# Supplementary material for: Multi-modal sensor fusion towards three-dimensional airborne sonar imaging in hydrodynamic conditions
Source: Commun Eng. 2023 Apr 25;2:16. doi: 10.1038/s44172-023-00065-4 (PMC10955972; doi:10.1038/s44172-023-00065-4)
Supplement: Supplementary file 2 — Supplementary Information [file 44172_2023_65_MOESM2_ESM.pdf]

Supplementary Information for

Multi-modal sensor fusion towards three-dimensional airborne sonar imaging in hydrodynamic conditions

Aidan Fitzpatrick, Roshan P. Mathews, Ajay Singhvi, and Amin Arbabian

March 2023

Supplementary Note 1: Maximum Imaging Depth

In conventional sonar imaging systems, the maximum range at which a target can be detected or imaged can be estimated using the *Sonar Equation*[1]:

$$SNR = SL - 2TL + TS - NL + AG \quad [dB], \tag{1}$$

where SNR is the signal-to-noise ratio in decibels, SL is the source level, TL is the transmission loss, TS is the target strength, NL is the noise level, and AG is the array gain. To find the maximum range from Equation (1), a detection threshold for the SNR can be assumed and the maximum tolerable transmission loss can be calculated.

To estimate the maximum imaging depth of the proposed photoacoustic airborne sonar system (PASS), we can employ a similar equation; however, the Sonar Equation cannot be directly used due to underlying assumptions of the sonar imaging geometry. For example, the factor of 2 on the transmission loss in Equation (1) is a result of the assumption that the source and receivers are co-located. In PASS, the acoustic source (generated by the laser) and the acoustic receivers (the CMUTs) are not even in the same medium.

Below we derive the *PASS Equation*, which considers the multi-physics excitation as well as the acoustic propagation through both water and air to provide an estimate of the maximum imaging depth. To simplify the derivation, we assume hydrostatic conditions. Equation symbols introduced throughout the derivation are defined in Supplementary Table I.

As presented in our previous work[2], the magnitude of the pressure generated by an intensity modulated laser is given by:

$$P(\omega) = \frac{T\omega\beta P_0 H(\omega)}{2C_p}\frac{\mu k}{\mu^2 + k^2}. \tag{2}$$

The source level of PASS can be calculated as the intensity of the acoustic source in decibels:

$$SL_{PASS} = 10\log_{10}\left(\frac{P(\omega)^2}{Z_{water}}\right). \tag{3}$$

The generated signal then incurs a transmission loss as it propagates to a depth z:

$$TL^{(i)}_{PASS} = \alpha_w(\omega)z + 10\log_{10}(z^2), \tag{4}$$

where the total transmission loss is the sum of the attenuation loss and the spreading loss.

At a depth z, the signal is incident on an acoustic scatterer with target strength:

$$TS_{PASS} = 10\log_{10}\left(\frac{I_r}{I_i}\right), \tag{5}$$

where the target strength is the ratio of the reflected signal,I_r, to the incident signal, I_i, at a distance 1 meter from the scattering object. The target strength is a function of many factors including the area of the scatterer, the material of the scatterer, the acoustic wavelength, and the directivity of the scattered pressure wave[1].

The reflected signal then propagates back up toward the water surface and through the air-water interface where it is detected by the airborne acoustic transducers. The transmission loss incurred by the reflected signal is:

$$TL^{(r)}_{PASS} = \alpha_w(\omega)z + \alpha_a(\omega)h + 10\log_{10}(z^2) - 10\log_{10}(\tau), \tag{6}$$

where the total transmission loss is the sum of the attenuation losses, the spreading loss, and the acoustic interface loss. The acoustic interface loss is a result of the acoustic impedance mismatch between air and water and is defined as:

$$\tau = 1 - \left(\frac{Z_{air} - Z_{water}}{Z_{air} + Z_{water}} \right)^2 . \tag{7}$$

Note that Equation (6) does not contain a term for the spreading loss in air; this is a reasonable approximation that holds due to the refraction of acoustic waves towards the surface normal as they transmit through the interface[3].

Acoustic transducers, including CMUTs, are often characterized in terms of their noise equivalent pressure (NEP)[4,5], which can be converted to noise level:

$$NL_{PASS} = 10 \log_{10} \left(\frac{NEP^2}{Z_{air}} \right) . \tag{8}$$

Lastly, under the assumption that the noise in the system is incoherent, the array gain is a result of the coherent integration of the signals captured by an array of N transducers:

$$AG_{PASS} = 10 \log_{10}(N). \tag{9}$$

The SNR of PASS can therefore be written as:

$$SNR_{PASS} = SL_{PASS} - TL^{(i)}_{PASS} + TS_{PASS} - TL^{(r)}_{PASS} - NL_{PASS} + AG_{PASS} \quad [dB]. \tag{10}$$

Using the derived PASS Equation, we can understand how the proposed system can scale to imaging greater water depths. To calculate the maximum depth, we assume a required SNR of 10 dB. A summary of other parameter values is displayed in Supplementary Table I.

Supplementary Fig. 1a illustrates the achievable imaging depth as a function of the peak laser power and the target strength. As mentioned above, the target strength is a function of many parameters and therefore is a complex topic; we refer the interested reader to additional resources[1,6] for more detail. That being said, a general rule-of-thumb is that larger targets tend to have higher target strengths. It is even possible, and not unusual, for a target to have a positive-valued target strength; this does not suggest that the reflected signal is greater than the incident signal but rather that the reflected signal has a degree of directivity[1]. To provide a few grounding examples: the target strength of a 25 cm fish is on the order of -35 dB whereas the target strength of a whale can be as high as 10 dB[6]. A common reference point is that an ideal reflecting sphere with a 2 meter radius ($a = 2$ m) has a target strength of 0 dB.

Supplementary Fig. 1b plots SNR as a function of the imaging depth while assuming a laser excitation with 50 kW peak power. The plot contains 5 traces for various target strengths. To enable further understanding of the system capabilities, we provide (as part of the supplemental code) a MATLAB script which allows input of user-defined parameters to generate alike figures.

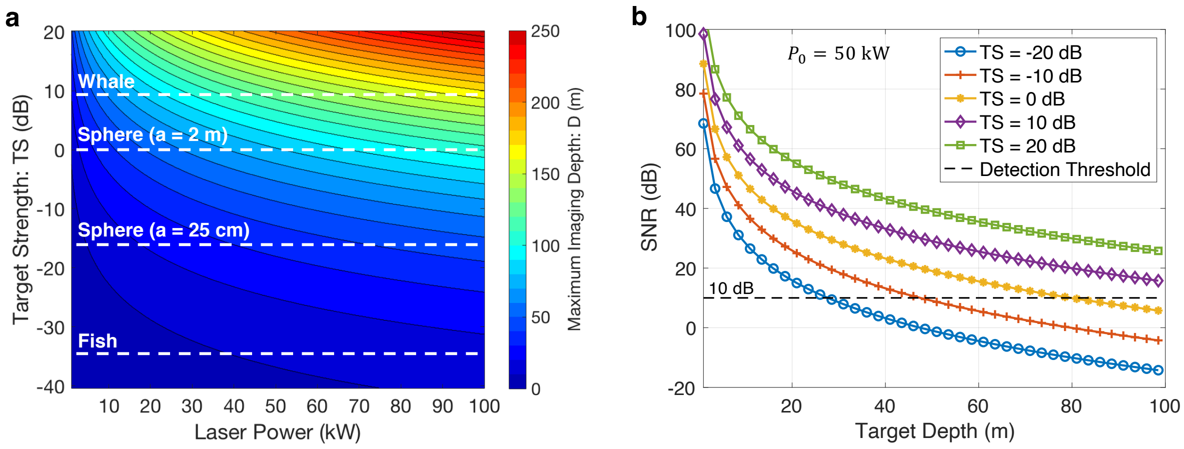

Supplementary Figure 1: **Maximum imaging depth found via the PASS Equation. a** Maximum imaging depth as a function of the target strength and peak laser power; each contour is a 10 m increment. **b** SNR of PASS as a function of the imaging depth for various target strengths ($P_0 = 50$ kW).

Supplementary Table I: Definition of PASS equation symbols and their assumed values.

Symbol	Definition	Assumed Value
P	spectral magnitude of the acoustic pressure	—
ω	angular acoustic frequency	$2\pi \cdot 50$ kHz
T	optical transmission coefficient[7]	0.98
β	thermal expansion coefficient of water[8]	2.5×10^{-4} 1/K
C_p	specific heat capacity of water[8]	4000 J/(kg·K)
P_0	peak laser power	0 - 100 kW
$H(\omega)$	normalized optical modulation function	—
k	acoustic wavenumber in water	209 rad/m
μ	optical absorption coefficient of water	209 1/m
Z_{water}	acoustic impedance of water[9]	1.5×10^6 Rayl
Z_{air}	acoustic impedance of air[9]	420 Rayl
z	water depth	—
$\alpha_w(\omega)$	frequency dependent acoustic attenuation in water[10]	0.015 dB/m (at 50 kHz)
$\alpha_a(\omega)$	frequency dependent acoustic attenuation in air[11]	1.73 dB/m (at 50 kHz)
h	height of the airborne transducers	3 m
NEP	noise equivalent pressure of the transducers	100 μPa
N	number of acoustic receivers in the array	1024

Comments on assumed values: The assumed frequency of 50 kHz is a trade-off between low frequency for minimizing attenuation losses and high frequency for increasing the photoacoustic conversion efficiency[2] and imaging resolution[3]; please refer to our previous work[2] for an analysis of the optimal acoustic frequency. The acoustic wavenumber is calculated for the assumed frequency of 50 kHz. The optical absorption coefficient is a function of the laser wavelength, and previously[2], we have shown that the optimal optical absorption coefficient is equal to the acoustic wavenumber; here we assume use of the appropriate laser wavelength (∼1200 nm) to achieve this optimum. A CMUT device with reasonably high sensitivity can have a NEP that is on the order of 100 μPa with recent devices reporting as low as 35 μPa NEP[4]. Lastly, we assume an acoustic receiver array with 1024 elements.

Supplementary Note 2: Surface Map Processing Pipeline

To use our system in hydrodynamic conditions, we have conceived a multi-modal sensor fusion framework which employs optical imaging of the water surface to complement the acoustic measurements of the underwater scene. By imaging the water surface, we obtain the spatial distribution of the speed-of-sound in the acoustic propagation channel which can be used to correct for the distortions incurred by the acoustic signals as they cross through the air-water interface. To accurately model the acoustic propagation it is necessary that the imaged surface profile is within the accuracy requirement stipulated in the main text. In practice, depth sensors are prone to dropouts and noise that manifest as holes, discontinuities and high spatial frequencies (see Supplementary Fig. 2a) that do not naturally exist in water waves. To remove such surface mapping artifacts, a dual stage signal processing pipeline is used to obtain a refined surface map which more accurately models the spatial distribution of the speed-of-sound, $c(x, y, z)$.

Stage 1 : Surface Interpolation - removing holes and discontinuities

Due to dropouts and noise, several points on the surface map have null readings and discontinuities. We define a discontinuity (or outlier) as a voxel in the surface map which has a depth reading that differs from the local median by one median absolute deviation[12]. A local moving window filter interpolates the discontinuous and null voxels with the value of the nearest neighboring non-outlier. The interpolated surface map without the holes and discontinuities is shown in Supplementary Fig. 2b-c in 2D and 3D, respectively. While replacing the outliers results in a continuous surface, the surface profile still contains high spatial frequencies that do not naturally exist. Consequently, an additional filtering stage is employed.

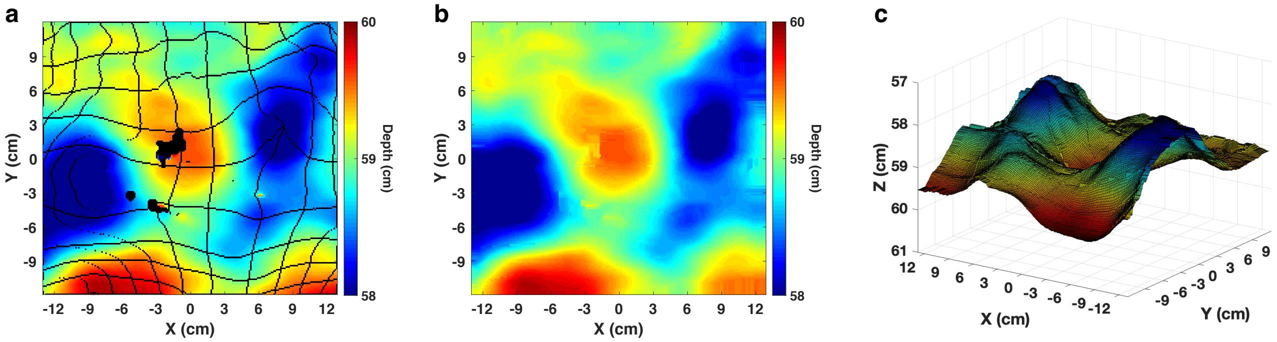

Supplementary Figure 2: **Interpolation.** **a** The raw depth data obtained from the depth sensor is contaminated with dropouts (null depth readings displayed in black) and noise making it unfit for direct use in modeling the acoustic channel. With surface interpolation we remove these holes and discontinuities from the surface map to get a closer representation of the true surface of water – shown in 2D in **b** and 3D in **c**.

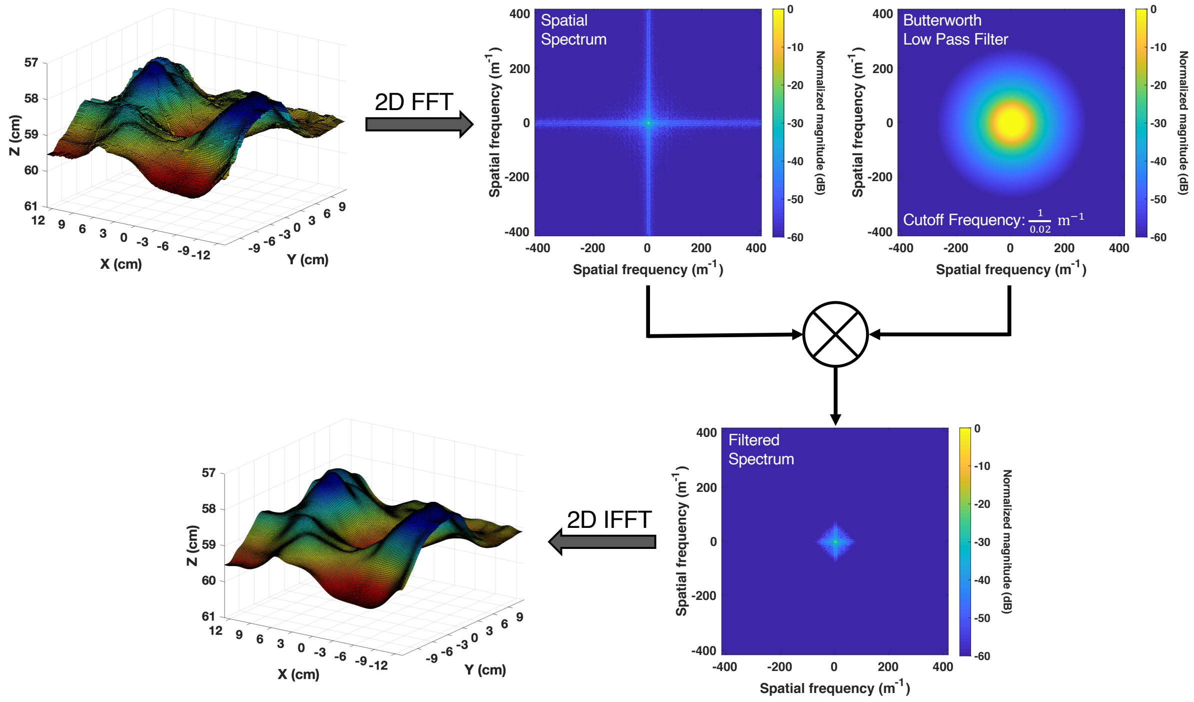

Supplementary Figure 3: **Filtering.** To remove residual artifacts with high spatial frequencies after Stage 1, low-pass filtering in the spatial frequency domain can preserve the longer wavelengths while attenuating wavelengths smaller than 2 cm (which are known to have negligible amplitudes).

Stage 2 : Filtering - removing high spatial frequencies

As articulated in the main text, water waves with wavelengths smaller than 2 cm have negligible amplitude. Therefore, we consider wavelengths smaller than 2 cm to be surface mapping artifacts. To suppress these artifacts, we perform filtering of the surface map in the spatial frequency domain as depicted in Supplementary Fig. 3.

First, we transform the surface map (after Stage 1) to the spatial frequency domain by performing a two-dimensional Fast Fourier Transform (2D FFT) over the X and Y dimensions. Suppressing small wavelengths is equivalent to filtering high spatial frequencies. We use a 4th order low-pass Butterworth filter which provides significant attenuation of high spatial frequencies while preserving the magnitude of the lower spatial frequencies. The Butterworth filter has a cutoff frequency (f_c) corresponding to a wavelength (L) of 2 cm where: $f_c = 1/L$ or 50 m^{-1}. In the spatial frequency domain, filtering is performed by multiplying the spatial spectrum of the surface map with the defined Butterworth low-pass filter. Finally, the filtered spectrum is transformed back to spatial domain using an Inverse FFT (2D IFFT). It is this resulting surface map from the described dual stage post-processing which can now be used to accurately model the acoustic propagation channel in the GPW-SAR algorithm.

Note: Please refer to the Supplementary Movie for a post-processed temporally varying surface wave profile that has been captured experimentally.

Supplementary Note 3: Speed-of-Sound Accuracy in the Channel Model

In the main manuscript, the presented multi-modal sensor fusion framework proposes mapping the surface of water in order to obtain an accurate model of the acoustic propagation channel. With an accurate channel model, the distortions incurred by the acoustic echoes as they cross through the non-planar air-water interface can be inverted by the presented image reconstruction algorithm. As part of the pre-processing pipeline in the sensor fusion framework, the acquired surface map is converted to a discretized 3D volumetric representation of the acoustic channel defined over space, $c(x, y, z)$, where voxels above the water surface are assigned the speed-of-sound in air, c_{air}, and voxels beneath the water surface are assigned the speed-of-sound in water, c_{water}.

In the Discussion section of the main text, we discuss the specifications for the required accuracy, spatial resolution, and frame rate of the surface mapping imager; however, another important aspect is the accuracy of c_{air} and c_{water} assumed in the channel model. For the experimental results presented in the main manuscript, $c_{air} = 340$ m/s and $c_{water} = 1500$ m/s were assumed.

Here, we evaluate the impact of incorrectly assumed speed-of-sound in the channel model. Specifically, since the proposed system concept is entirely airborne, we focus on an incorrectly modeled c_{water} as c_{air} can be directly measured by the airborne system[13]. An important note regarding the GPW-SAR algorithm is the fact that once the acoustic signals are migrated through the air-water interface (i.e. Steps 4-5 presented in main manuscript), the reconstruction in the water becomes equivalent to a classic in-water sonar reconstruction problem. This issue of unknown speed-of-sound as a function of water depth is well-studied and well-modeled in the sonar literature, and thus future iterations of the GPW-SAR algorithm can leverage decades of research on techniques and auto-correcting algorithms that help to solve this problem[14,15].

Consequently, it is most important that our modeled speed-of-sound has minimal error at the water surface so that the distortions can be appropriately compensated prior to employing existing techniques. While the proposed system cannot directly measure c_{water} at the surface in-situ, many works have developed models for the speed-of-sound in water as a function of key parameters including the water temperature (T), the water salinity (S), and the water depth (D). For example, Medwin provided one such equation which reduces more advanced models to a simplified equation with negligible loss of precision[16]:

$$c_{water} = 1449.2 + 4.6T - 0.055T^2 + 0.00029T^3 + (1.34 - 0.01T)(S - 35) + 0.016D \quad [m/s]. \tag{11}$$

As can be seen in Equation (11), the water temperature has the greatest impact on the speed-of-sound near the water surface. Ocean water salinity typically varies from about 33-37 ppt and thus has a minimal impact on the speed-of-sound in water; therefore, assuming $S = 35$ ppt, we calculate the derivative of c_{water} with respect to temperature:

$$\frac{\partial c_{water}}{\partial T} = 4.6 - 0.11T + 0.00087T^2. \tag{12}$$

Through Equation (12), if we evaluate the uncertainty in c_{water} at $T = 20°$C (i.e. a typical value), we realize the uncertainty to be ± 2.75 m/s for every ± 1°C temperature uncertainty. It is also important to note that there are many approaches for measuring the sea surface temperature remotely that could allow us to measure the surface temperature from our airborne system with reasonably high accuracy[17–19]. Even with a poor measurement accuracy of surface temperature with ± 5°C uncertainty, c_{water} is expect to be in the range 1510 - 1540 m/s.

Now, we can utilize the simulation described in the Surface Mapping Accuracy subsection of the Discussion section in the main manuscript to evaluate the error on the signals migrated through the air-water interface using the GPW-SAR algorithm with an incorrectly modeled c_{water}. The algorithm is run over the range 1510 - 1540 m/s, where the ground-truth simulated value is $c_{water} = 1525$ m/s. As shown in Supplementary Fig. 4a, we perform the GPW-SAR reconstruction in two steps where first, we migrate the received signals to just beneath the water surface (from (1) to (2)), where we evaluate the normalized root-mean square error (NRMSE):

$$NRMSE = \frac{||s||_2 - ||\hat{s}||_2}{||s||_2} \tag{13}$$

where s is the vector of migrated signals using the ground-truth speed-of-sound value, $\hat{s}$ is the vector of migrated signals using the incorrect speed-of-sound value, and $|| \cdot ||_2$ is the L2-norm.

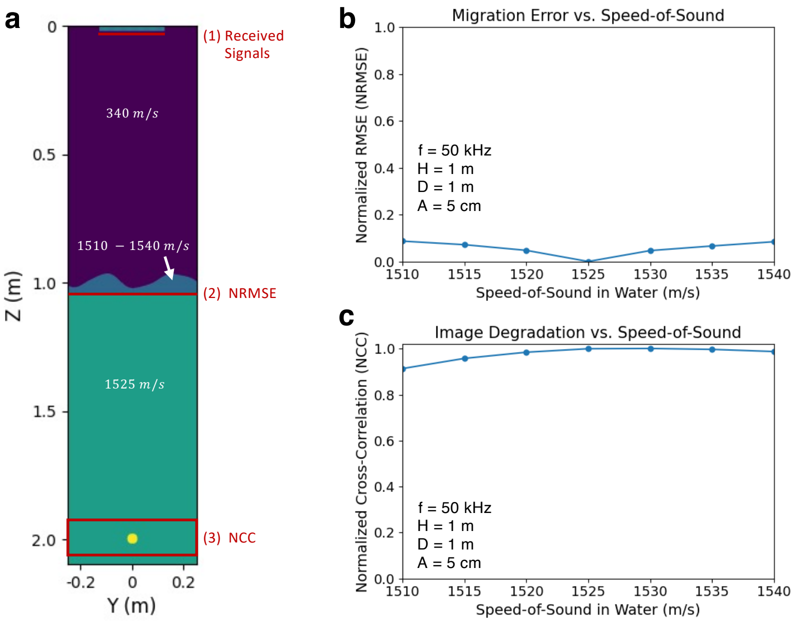

Supplementary Figure 4: **Effect of Speed-of-Sound Accuracy.** **a** Simulation and reconstruction setup showing that first, the received signals are migrated to just beneath the water surface where the normalized root-mean square error (NRMSE) is calculated, and then, those migrated signals are used to reconstruct the image where the normalized cross-correlation (NCC) is calculated. Both metrics are normalized with respect to the ground-truth. The simulation uses an acoustic frequency $f = 50$ kHz, receiver height $H = 1$ m, target depth $D = 1$ m, and wave height $A = 5$ cm. **b** Calculated NRMSE just beneath the water surface as a function of the assumed speed-of-sound. **c** Calculated NCC of the reconstructed image for the signals that were migrated to beneath the water surface over the range of assumed speed-of-sound.

Next, we complete the image reconstruction in the water (from (2) to (3)) assuming the ground-truth value of $c_{water} = 1525$ m/s. This two-step process isolates the impact of imperfect distortion correction across the air-water interface assuming the remainder of the reconstruction can be performed optimally using an auto-correcting algorithm as mentioned above.

The results of this analysis are shown in Supplementary Fig. 4b-c. First, in Supplementary Fig. 4b, it can be seen that the NRMSE is low across the entire range of speed-of-sounds evaluated; however, this shows that errors are incurred as a result of imperfect distortion correction. In Supplementary Fig. 4c, we evaluate the degradation in image quality to understand the impact of imperfect distortion correction. It can be seen that the degradation in image quality is relatively low over this range of incorrectly modeled c_{water}, thus demonstrating that remotely measuring the water temperature could be used to obtain image reconstructions in-line with those expected from conventional in-water sonar systems – assuming robustness of models akin to that provided in Equation (11).

Supplementary References

1. Urick, R. *Principles of Underwater Sound* ISBN: 9780070660878. `https://books.google.com/books?id=hfxQAAAAMAAJ` (McGraw-Hill, 1983).
2. Fitzpatrick, A., Singhvi, A. & Arbabian, A. An airborne sonar system for underwater remote sensing and imaging. *IEEE Access* **8,** 189945–189959 (2020).
3. Singhvi, A., Fitzpatrick, A. & Arbabian, A. *Resolution Enhanced Non-Contact Thermoacoustic Imaging using Coded Pulse Excitation* in *2020 IEEE International Ultrasonics Symposium (IUS)* (2020), 1–4.
4. Ma, B., Firouzi, K., Brenner, K. & Khuri-Yakub, B. T. *High sensitivity and wide bandwidth airborne CMUTs with low driving voltage* in *2019 IEEE International Ultrasonics Symposium (IUS)* (2019), 1201–1204.
5. Ma, B., Firouzi, K., Brenner, K. & Khuri-Yakub, B. T. Wide bandwidth and low driving voltage vented CMUTs for airborne applications. *IEEE transactions on ultrasonics, ferroelectrics, and frequency control* **66,** 1777–1785 (2019).
6. Hodges, R. P. *Underwater acoustics: Analysis, design and performance of sonar* (John Wiley & Sons, 2011).
7. Hale, G. M. & Querry, M. R. Optical constants of water in the 200-nm to 200-μm wavelength region. *Applied optics* **12,** 555–563 (1973).
8. Feistel, R. APPENDIX TO EOLSS ARTICLE 02-03-07 THERMODYNAMIC PROPERTIES OF SEAWATER.

9. Filippi, P., Bergassoli, A., Habault, D. & Lefebvre, J. P. *Acoustics: basic physics, theory, and methods* (Elsevier, 1998).

10. Francois, R. & Garrison, G. Sound absorption based on ocean measurements. Part II: Boric acid contribution and equation for total absorption. *The Journal of the Acoustical Society of America* **72,** 1879–1890 (1982).

11. Bass, H. E., Sutherland, L. C., Zuckerwar, A. J., Blackstock, D. T. & Hester, D. Atmospheric absorption of sound: Further developments. *The Journal of the Acoustical Society of America* **97,** 680–683 (1995).

12. Hampel, F. R. The influence curve and its role in robust estimation. *Journal of the american statistical association* **69,** 383–393 (1974).

13. Chande, P. & Sharma, P. A fully compensated digital ultrasonic sensor for distance measurement. *IEEE Transactions on instrumentation and measurement* **33,** 128–129 (1984).

14. Wahl, D. E., Eichel, P., Ghiglia, D. & Jakowatz, C. Phase gradient autofocus-a robust tool for high resolution SAR phase correction. *IEEE Transactions on Aerospace and electronic systems* **30,** 827–835 (1994).

15. Piper, J. E. & Sternlicht, D. D. *A low-order autofocus algorithm* in *OCEANS'11 MTS/IEEE KONA* (2011), 1–4.

16. Medwin, H. Speed of sound in water: A simple equation for realistic parameters. *The Journal of the Acoustical Society of America* **58,** 1318–1319 (1975).

17. Zhang, K. & Wang, X. High-Precision Measurement of Sea Surface Temperature with Integrated Infrared Thermometer. *Sensors* **22,** 1872 (2022).

18. Donlon, C. *et al.* An infrared sea surface temperature autonomous radiometer (ISAR) for deployment aboard volunteer observing ships (VOS). *Journal of Atmospheric and Oceanic Technology* **25,** 93–113 (2008).

19. Saunders, P. M. Aerial measurement of sea surface temperature in the infrared. *Journal of Geophysical Research* **72,** 4109–4117 (1967).
