# Supplementary material for: Multi-modal sensor fusion towards three-dimensional airborne sonar imaging in hydrodynamic conditions
Source: Commun Eng. 2023 Apr 25;2:16. doi: 10.1038/s44172-023-00065-4 (PMC10955972; doi:10.1038/s44172-023-00065-4)
Supplement: Supplementary file 3 — Description of Additional Supplementary File [file 44172_2023_65_MOESM3_ESM.pdf]

Description of Additional Supplementary File

File name: Supplementary Movie

Description: Depiction of post-processed surface waves. This movie illustrates an experimentally captured time-varying surface wave after post-processing of the raw coded light depth measurements.
